# Supplementary material for: Filming movies of attosecond charge migration in single molecules with high harmonic spectroscopy
Source: Nat Commun. 2022 Aug 6;13:4595. doi: 10.1038/s41467-022-32313-0 (PMC9357086; doi:10.1038/s41467-022-32313-0)
Supplement: Supplementary file 1 — Supplementary Information [file 41467_2022_32313_MOESM1_ESM.pdf]

Supplementary Information for
Filming movies of attosecond charge migration in single molecules with high harmonic spectroscopy

Lixin He[1,2], Siqi Sun[1], Pengfei Lan[1,2], Yanqing He[1], Bincheng Wang[1], Pu Wang[1], Xiaosong Zhu[1,2], Liang Li[1,2], Wei Cao[1,2], Peixiang Lu[1,2,3], and C. D. Lin[4]

[1]Wuhan National Laboratory for Optoelectronics and School of Physics,
Huazhong University of Science and Technology,
Wuhan 430074, China

[2]Optical Valley Laboratory,
Hubei 430074, China

[3]CAS Center for Excellence in Ultra-intense Laser Science,
Shanghai 201800, China

[4]Department of Physics, Cardwell Hall,
Kansas State University,
Manhattan, KS 66506, USA

The Supplementary Information contains:

Supplementary Note 1: Experimental set-up

Supplementary Note 2: Multiple orbitals effect in HHG from CO_2 molecule

Supplementary Note 3: Comparison between the alignment-averaged and single-molecule harmonic dipoles

Supplementary Note 4: Reconstruction algorithm for molecular charge migration

Supplementary Note 5: Time-dependent density functional theory calculations

Supplementary Note 6: Influence of alignment average on the reconstruction

Supplementary Note 7: Field-free charge migration in N_2^+ and CO_2^+

Supplementary Figures 1-15

Supplementary References

Supplementary Note 1: Experimental set-up

Our experiment is carried out by using a commercial Ti: sapphire laser system (Legend Elite-Duo, Coherent, Inc.), which delivers 35-fs, 800-nm laser pulses at a repetition rate of 1 kHz. As illustrated in Supplementary Fig. 1, the output laser is split into two beams (P_1 and P_2) by a beam splitter (BS). One with moderate intensity (P_1) is used to induce nonadiabatic alignment of molecules along its polarization. The other intense one (P_2) provides a probe pulse to interact with the aligned molecules for high harmonic generation (HHG). In this work, we have performed experiments with both one-color and two-color driving scheme. In the one-color experiment, the P_2 pulse is directly used to generate high harmonics. In the two-color experiment, the P_2 pulse is remolded to a parallel two-color driving field. Specifically, a type-I BBO crystal is used to produce a second harmonic (SH) field of the 800 nm fundamental laser. The group-velocity dispersion between the SH and fundamental fields is compensated using a calcite plate. The relative phase between the SH and fundamental fields is controlled by a pair of wedges. A wire grid polarizer (WGP) is used to ensure the same polarizations of the SH and fundamental fields. The relative intensity between the SH and the fundamental fields is controlled by the combination of a dual-wave plate (DWP, a half wavelength plate at 800-nm and full wavelength plate at 400-nm) and the WGP. In our experiment, the intensity of the SH field is about 2×10^{-3} of the fundamental field. The 800-nm laser intensity has been estimated from the harmonic cutoff, which is about 1.95×10^{14} W/cm^2 in the one-color experiment and 1.5×10^{14} W/cm^2 in the two-color experiment. In both the one-color and two-color experiments, the alignment and probe pulses are parallel in polarization. A motorized delay line is installed in the arm of the alignment pulse to adjust the time delay between the two pulses. These two pulses are collinearly focused into a supersonic gas jet ejected from a nozzle (250 μm diameter) with a backing pressure of 0.8 bars by a spherical mirror (f=250 mm). The gas jet is placed 2 mm after the laser focus to ensure good phase matching of the short-trajectory harmonics. The high harmonics generated by the probe pulse is detected by a homemade flat-field soft x-ray spectrometer [1], which consists of a 0.1-mm-wide, 15-mm-height entrance slit, a flat-field grating (1200 grooves mm^{-1}), and a microchannel plate (MCP) backed with a phosphor screen. A charge-coupled device (CCD) camera is used to record the spectral images.

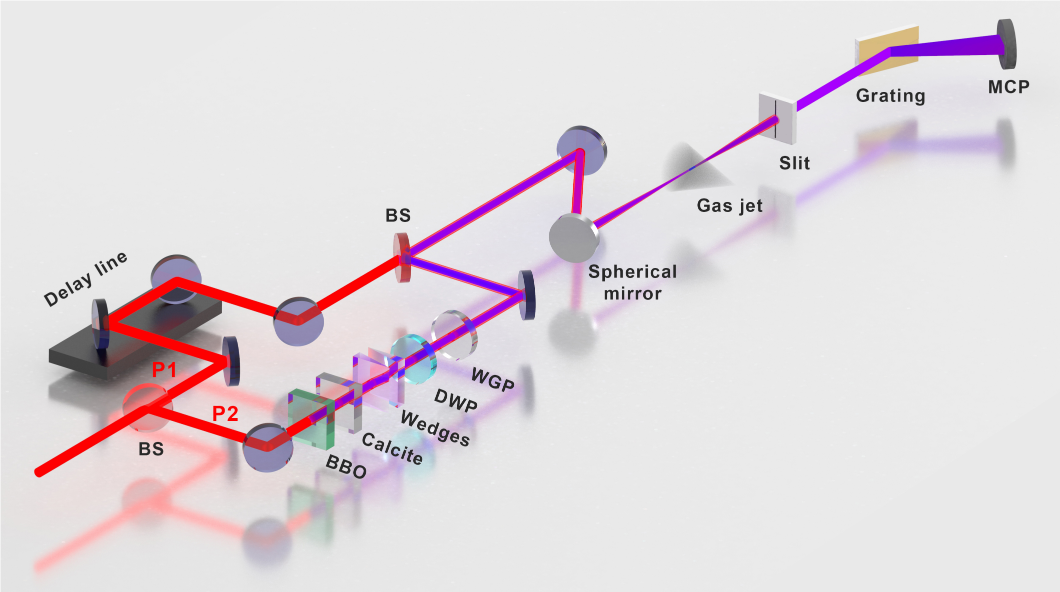

Supplementary Fig. 1: **Sketch of the experimental set-up.** The output of a commercial Ti: sapphire is split into two beams P_1 and P_2. The pulse P_1 is used to align the molecules along its polarization. The pulse P_2 is used directly (one-color scheme) or remolded to a parallel two-color laser field (two-color scheme) to interact with the aligned molecules to generate high-order harmonics. The generated harmonics are detected by a homemade flat-field soft x-ray spectrometer [including an entrance slit, a flat-field grating, and a microchannel plate (MCP)]. BS: beam splitter, DWP: dual-wave plate, WGP: wire grid polarizer.

Supplementary Note 2: Multiple orbitals effect in HHG from CO_2 molecule

The multiple orbitals effect in HHG from N_2 have been shown in the main text. Here, we demonstrate that similar results can also be found in HHG from CO_2 molecule. Supplementary Figs. 2(a)-(c) display the alignment-angle-dependent harmonic spectra from HOMO, HOMO-1 and HOMO-2 of CO_2 calculated with the quantitative rescattering (QRS) theory [2,3]. As shown in Supplementary Fig. 2(a), HHG from the HOMO orbital of CO_2 presents a double-hump structure as the alignment angle varies. The small hump is around $\theta = 25°$ and the big one is around $\theta = 70°$. After averaging over molecular alignment distribution, the angular dependence of the HHG intensity will become smoother, and show a maximum near $\theta = 90°$ [2,3]. For the HOMO-1 orbital of CO_2, the HHG intensity is more pronounced for larger alignment angles due to its Π_u symmetry. The situation is opposite for the HOMO-2 orbital, of which the HHG intensity is maximized around $\theta = 0°$ due

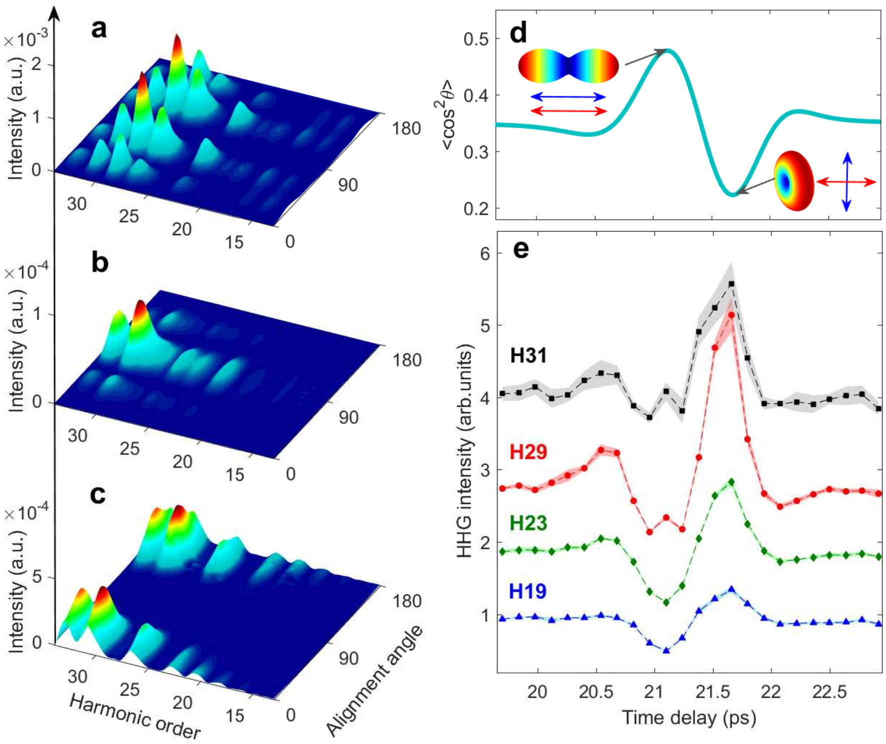

Supplementary Fig. 2: **Harmonic generation from CO_2 molecule.** (a)-(c) Theoretically calculated alignment-angle-dependent harmonic spectra from the HOMO, HOMO-1 and HOMO-2 orbitals of CO_2 molecule. Here, the calculations are performed with one-color laser parameters. (d) Time-dependent degrees of molecular alignment $\langle\cos^2\theta\rangle(t)$ around the half rotational revival of CO_2 in our experiment. In the CO_2 experiment, the intensity of the alignment pulse is estimated to be 4×10^{13} W/cm^2, and the rotational temperature is about 100 K. The embedded false-color plots with prolate and oblate shapes correspond to the angular distributions of molecules at the alignment (21.1 ps) and anti-alignment (21.66 ps) moments, respectively. Blue and red arrows indicate the directions of molecular alignment and the polarization of the driving laser. (e) Measured HHG signals of H19, H23, H29 and H31 of CO_2 at the time delays in (d). Here, the harmonic intensities have been normalized to results measured for the isotropic case. The results of H23, H29 and H31 have been shifted vertically.

to its Σ_u symmetry. Moreover, both the HOMO-1 and HOMO-2 contributions are more appreciable for higher order harmonics, e.g, H27-H31, due to their larger cross sections in high energy range. We have also performed HHG experiment with CO_2 molecule. In the experiment, the polarizations of the alignment and probe pulses are parallel. Supplementary

Figure 2(d) plots the time-dependent alignment degree calculated under the experimental conditions. Supplementary Figure 2(e) shows the harmonic signals measured near the half rotational revival of CO_2 molecule. One can see that, for lower-order harmonics, e.g., H19 and H23, the time-dependent harmonic intensities present inverted modulations compared to the curve of $\langle\cos^2\theta\rangle(t)$. In detail, the harmonic intensity shows a minimum (maximum) at the time delay of 21.1 ps (22.66 ps), where the molecules are aligned (anti-aligned) with a prolate (oblate) distribution in the direction parallel (perpendicular) to the polarization of the probe pulse. These results agree with the features of HOMO and HOMO-1 of CO_2. However, due to the much weaker ionization rate of HOMO-1 at small alignment angles, the HOMO-1 contribution to HHG is usually ignored for a parallel polarization configuration of the alignment and probe pulses in previous works [4,5]. In this work, our reconstruction reveals a considerable population of the $\tilde{A}$ state (HOMO-1) at $\theta = 0^\circ$ [see Fig. 2(e) in the main text] due to the remarkable laser-induced transition between the $\tilde{X}$ and $\tilde{A}$ states of CO_2^+ ion (see Supplementary Note 5 below). For much higher order harmonics, e.g., H29 and H31, the minimum at 21.1 ps is gradually flattened, indicating substantial contribution of HOMO-2 in the high energy range.

Supplementary Note 3: Comparison between the alignment-averaged and single-molecule harmonic dipoles

Molecular alignment, an efficient way to connect the molecular and laboratory frames, plays a significant role in molecular HHG experiment. However, in experiment, the molecular ensemble can never be perfectly aligned. The measurement is a coherent superposition of the individual emission from molecules aligned at different angles. Here, we demonstrate that imperfect molecular alignment in experiment will dramatically make the measurement deviate from the single-molecule response. In HHG experiment, the measured dipole moment for HHG is an average of the single-molecule response weighted by the alignment distribution [6,7], i.e.,

$$D_{\rm ave}(\vartheta) = \int_0^{\pi}\int_0^{2\pi} D[\Theta(\theta,\phi,\vartheta)]\rho(\theta) sin\theta d\theta d\phi, \tag{S1}$$

where θ and ϕ are the polar and azimuthal angles of molecular axis. $\rho(\theta)$ is the molecular axis distribution. Imposed by the linearly polarized alignment pulse, the resulting molecular rotational wave packet has a cylindrical symmetry in space, thus the molecular axis

distribution $\rho(\theta)$ is independent of the azimuthal angle ϕ [8,9]. ϑ is the angle between the polarizations of the alignment and probe pulses. Θ is the angle between the molecular axis and the polarization of the probe pulse, which obeys $\cos\Theta=\sin\theta\sin\vartheta\cos\phi+\cos\theta\cos\vartheta$. Specially, for a parallel polarization of these two pulses (i.e., ϑ=0), $\Theta=\theta$. D is the single-molecule dipole moment. In the following, we use the dipole moment of H21 retrieved from the two-color experiment of N_2 molecule [see Supplementary Fig. 7(a) below] to calculate the measured dipole moment D_{ave}.

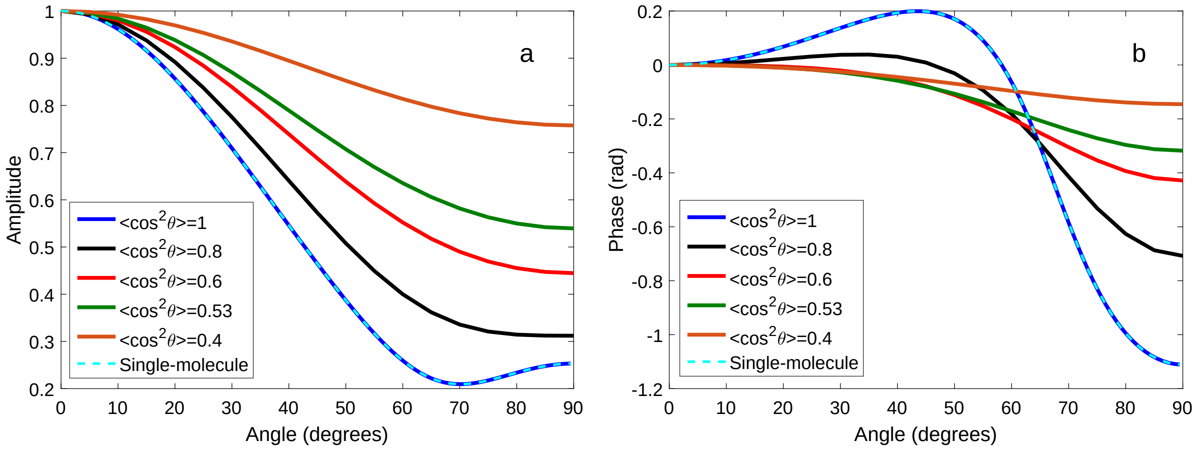

Supplementary Fig. 3: **Comparison between the alignment-averaged and single-molecule harmonic dipoles.** (a)-(b) show the angle-resolved dipole amplitudes and phases calculated for different molecular alignment degrees (solid lines). For comparison, the single-molecule results are also presented as the dashed lines. In (a), the angle-dependent dipole amplitudes have been normalized by the result at 0°. (b) shows the angle-dependent relative phases with respect to that at 0°.

In Supplementary Fig. 3, we plot the angle-dependent dipole moment $D_{\text{ave}}(\Theta)$ (both amplitude and phase) measured by scanning the angle between the alignment and probe pulses for different molecular alignment degrees (solid lines). For comparison, the single-molecule results are also presented (dashed lines). As shown, the measured dipole amplitudes [Supplementary Fig. 3(a)] and phases [Supplementary Fig. 3(b)] can coincide with the single-molecule results (dashed lines) only in the case of $\langle\cos^2\theta\rangle$=1 (blue solid lines), i.e., the case of perfect alignment. For the cases of imperfect alignment, e.g., $\langle\cos^2\theta\rangle$=0.4 (orange solid lines), 0.53 (green solid lines, the case in our experiment), 0.6 (red solid lines), and 0.8 (black solid lines), the measurements deviate from the single-molecule results. Moreover, the deviation

increases dramatically with the decrease of the degree of molecular alignment. The coherent average over molecular alignment distribution induced by the inevitable imperfect molecular alignment in experiment could severely blur the single-molecule information, which in turn will reduce or even destroy the accuracy of the reconstruction. Thus, disentangling this internal angular coherence and achieving a true measurement of the single-molecule harmonic response is crucial for HHS.

Supplementary Note 4: Reconstruction algorithm for molecular charge migration

In experiment, HHG from the aligned molecule ensemble is a coherent superposition of the individual emission from molecules distributed at different angles. For a given harmonic, the time-dependent HHG intensity is given by [6,7]

$$S(\tau) = \left| \int_0^{\pi} D(\theta)\rho(\theta,\tau)\sin\theta d\theta \right|^2, \tag{S2}$$

where θ is the alignment angle of the molecule, and $\rho(\theta,\tau)$ is the molecular axis distribution. $D(\theta)$=$|D(\theta)|$exp$[i\varPhi(\theta)]$ is the alignment-dependent dipole moment of HHG on single-molecule level, which can be expressed as a coherent superposition of the induced dipole moment of each emission channel, i.e.,

$$D(\omega,\theta) = \sum_i D_i(\omega,\theta). \tag{S3}$$

Here $D_i(\omega,\theta)$ is the induced dipole moment related to each emission channel, which can be expressed as

$$D_i(\omega,\theta) = d^i_{\text{ion}}(\omega,\theta)a^i_{\text{acc}}(\omega)d^i_{\text{rec}}(\omega,\theta), \tag{S4}$$

where

$$d^i_{\text{ion}} = \sqrt{\eta_i}\langle\Psi_0(N)|\tilde{D}|\Phi_i(N-1)\chi_k\rangle \tag{S5a}$$

$$= \sqrt{\eta_i}\langle\psi_i|r|\chi_k\rangle \tag{S5b}$$

is the ionization transition dipole moment. η_i is the ionization rate of each channel. $a^i_{\text{acc}}(\omega)$ denotes the propagation amplitude of the electron wave packet in the continuum. In Eq. (S5a), $\Psi_0(N)$ is the ground state of the N-electron molecule, $\Phi_i(N-1)$ is the ground

state or excited state of the (N-1)-electron molecular ion, where a molecular orbital ψ_i has been removed to a continuum state χ_k. Both many-electron wavefunctions $\Psi_0(N)$ and $\Phi_i(N-1)\chi_k$ are properly antisymmetrized and $\tilde{D}$ is the dipole operator from all the electrons. In obtaining Eq. (S5b), we assume that all the molecular orbitals are in the neutral and the ion does not change before and after ionization.

To calculate the recombination transition dipole d^i_{rec}, we take into account that the molecular ion has been modified by the laser field during the time interval between ionization and recombination to $\Phi'_i(N-1)$, where

$$\Phi'_i(N-1) = \sum_j C_{ij}(\theta)\Phi_j(N-1). \tag{S6}$$

Thus, the recombination dipole can be written as

$$\begin{aligned} d^i_{\text{rec}} &= \langle \Phi'_i(N-1)\chi_k|\tilde{D}|\Psi_0(N)\rangle \\ &= \sum_j C_{ij}(\theta)\langle \chi_k|r|\psi_j\rangle. \end{aligned} \tag{S7}$$

Inserting Eq. (S7) to Eq. (S4), we obtain

$$D_i(\omega,\theta) = \sum_j C_{ij}(\theta) d^i_{\text{ion}}(\omega,\theta) a^i_{\text{acc}}(\omega) d^j_{\text{rec}}(\omega,\theta). \tag{S8}$$

With Eq. (S8), the total dipole moment for HHG then can be expressed as

$$D(\omega,\theta) = \sum_{ij} C_{ij}(\theta)\bar{D}_{ij}(\omega,\theta), \tag{S9}$$

where

$$\bar{D}_{ij}(\omega,\theta) = d^i_{\text{ion}}(\omega,\theta) a^i_{\text{acc}}(\omega) d^j_{\text{rec}}(\omega,\theta). \tag{S10}$$

In Eq. S9, the complex-valued coefficients $C_{ij}(\theta)$ are directly associated with the electron dynamics in the ionized molecule. Eqs. (S2) and (S9) set up a function between $S(\tau)$ and the coefficients $C_{ij}(\theta)$, making it possible to reconstruct these coefficients from the time-dependent harmonic signal $S(\tau)$. However, solving this inverse problem directly is rather difficult due to the extreme nonlinearity and that this function is ill-posed. In order to reduce the difficulty of reconstruction, we divide this problem into two steps: (1) decoupling $D(\theta)$ from $S(\tau)$, (2) retrieving $C_{ij}(\theta)$ from $D(\theta)$.

Step 1 is a nonlinear inverse problem. Here we introduce an intermediate quantity $R(\theta_1, \theta_2)$ [10,11] to linearize this problem. In detail, Eq. (S2) is first expanded as

$$S(\tau) = \left[\int_0^\pi D(\theta)\rho(\theta,\tau)\sin\theta d\theta\right]^* \left[\int_0^\pi D(\theta)\rho(\theta,\tau)\sin\theta d\theta\right]$$
$$= \int_0^\pi \int_0^\pi D^*(\theta_1)D(\theta_2)\rho(\theta_1,\tau)\rho(\theta_2,\tau)\sin\theta_1\sin\theta_2 d\theta_1 d\theta_2. \tag{S11}$$

Let

$$R(\theta_1,\theta_2) = \mathrm{Re}[D^*(\theta_1)D(\theta_2)], \tag{S12}$$

$$\rho(\theta_1,\theta_2,\tau) = \rho(\theta_1,\tau)\rho(\theta_2,\tau)\sin\theta_1\sin\theta_2 d\theta_1 d\theta_2, \tag{S13}$$

then $S(\tau)$ can be discretized as

$$S(\tau) = \sum_p \sum_q R(\theta_1^p, \theta_2^q)\rho(\theta_1^p, \theta_2^q, \tau). \tag{S14}$$

In Eq. (S12), the imaginary part of $D^*(\theta_1)D(\theta_2)$ vanishes after convoluting with a symmetric function $\rho(\theta_1,\theta_2,\tau)$ due to its asymmetry upon the exchange of θ_1 and θ_2. Thus in Eq. (S12), only the real part of $D^*(\theta_1)D(\theta_2)$ is kept. As mentioned in the main text, the molecular axis distribution $\rho(\theta,\tau)$ [therefore $\rho(\theta_1,\theta_2,\tau)$] can be determined from the time-dependent HHG signals in one-color experiment. Note that, the alignment conditions are the same for the one-color and two-color experiments.

To solve Eq. (S14), we develop a reconstruction algorithm by using a widely used ML algorithm—sparse representation [12]. Sparsity exists widely in the observations of physical world because the actual signals often contain certain fixed patterns that occupy only a small part of the representation space. Sparse representation recently has been used in many fields, e.g., signal processing [13], image processing [14], compressive sensing [15], PDE discovery [16], computer vision [17], and so on. In our work, sparse representation is the important foundation of the reconstruction algorithm. Assuming an observable vector $\mathbf{S}\in\mathbf{C}^n$ can be represented as a linear combination of a complete dictionary $\mathbf{D}\in\mathbf{C}^{n\times m}$ by $\mathbf{S}=\mathbf{D}\xi$, then the vector $\xi\in\mathbf{C}^m$ is called the representation of the observable $\mathbf{S}$ in the dictionary $\mathbf{D}$. Sparse representation problem is to find the possible representation ξ with the fewest non-zero elements. However, sometimes the representation of the observation vector in the

current dictionary is not sparse, so we need to construct a new dictionary to get a sparse representation of the observation vector in the new dictionary. In our case, Eq. (S14) can be rewritten as **S**=**QR** by defining

$$\mathbf{S} = \begin{pmatrix} S(\tau_1) \\ \vdots \\ S(\tau_k) \end{pmatrix}, \tag{S15}$$

$$\mathbf{R} = \begin{pmatrix} R(\theta_1^1, \theta_2^1) \\ \vdots \\ R(\theta_1^1, \theta_2^N) \\ R(\theta_1^2, \theta_2^1) \\ \vdots \\ R(\theta_1^2, \theta_2^N) \\ \vdots \\ R(\theta_1^N, \theta_2^N) \end{pmatrix}, \tag{S16}$$

$$\mathbf{Q} = \begin{pmatrix} \rho(\theta_1^1, \theta_2^1, t_1) & \cdots & \rho(\theta_1^1, \theta_2^N, t_1) & \rho(\theta_1^2, \theta_2^1, t_1) & \cdots & \rho(\theta_1^2, \theta_2^N, t_1) & \cdots & \rho(\theta_1^N, \theta_2^N, t_1) \\ \rho(\theta_1^1, \theta_2^1, t_2) & \cdots & \rho(\theta_1^1, \theta_2^N, t_2) & \rho(\theta_1^2, \theta_2^1, t_2) & \cdots & \rho(\theta_1^2, \theta_2^N, t_2) & \cdots & \rho(\theta_1^N, \theta_2^N, t_2) \\ \vdots & & \vdots & \vdots & & \vdots & & \vdots \\ \rho(\theta_1^1, \theta_2^1, t_k) & \cdots & \rho(\theta_1^1, \theta_2^N, t_k) & \rho(\theta_1^2, \theta_2^1, t_k) & \cdots & \rho(\theta_1^2, \theta_2^N, t_k) & \cdots & \rho(\theta_1^N, \theta_2^N, t_k) \end{pmatrix}. \tag{S17}$$

In the dictionary **Q**, the representation of the observation vector of **S** is **R**, which however doesn't have the sparsity. To get a sparse representation of **S**, we build a new dictionary matrix by expanding the **R** matrix with a series of two-dimensional Legendre polynomial

basis functions, i.e., $\mathbf{R}$=$\mathbf{F}\xi$, where

$$\mathbf{F} = \begin{pmatrix} B_{00}(\theta_1^1, \theta_2^1) & B_{20}(\theta_1^1, \theta_2^1) & B_{22}(\theta_1^1, \theta_2^1) & \cdots & B_{MM}(\theta_1^1, \theta_2^1) \\ \vdots & \vdots & \vdots & & \vdots \\ B_{00}(\theta_1^1, \theta_2^N) & B_{20}(\theta_1^1, \theta_2^N) & B_{22}(\theta_1^1, \theta_2^N) & \cdots & B_{MM}(\theta_1^1, \theta_2^N) \\ B_{00}(\theta_1^2, \theta_2^1) & B_{20}(\theta_1^2, \theta_2^1) & B_{22}(\theta_1^2, \theta_2^1) & \cdots & B_{MM}(\theta_1^2, \theta_2^1) \\ \vdots & \vdots & \vdots & & \vdots \\ B_{00}(\theta_1^2, \theta_2^N) & B_{20}(\theta_1^2, \theta_2^N) & B_{22}(\theta_1^2, \theta_2^N) & \cdots & B_{MM}(\theta_1^2, \theta_2^N) \\ \vdots & \vdots & \vdots & & \vdots \\ B_{00}(\theta_1^N, \theta_2^N) & B_{20}(\theta_1^N, \theta_2^N) & B_{22}(\theta_1^N, \theta_2^N) & \cdots & B_{MM}(\theta_1^N, \theta_2^N) \end{pmatrix} \tag{S18}$$

with

$$B_{mn}(\theta, \theta) = \frac{1}{\sqrt{2}}[L_m(\theta_1)L_n(\theta_2) + L_m(\theta_2)L_n(\theta_1)]. \tag{S19}$$

Here, $L_{m(n)}(\theta)$ is the normalized Legendre polynomial, M is the highest order of the Legendre polynomial. Due to the symmetry property of $R(\theta_1, \theta_2)$, only even m and n are included in Eqs. (S18)-(S19). Note that the choice of the transformation matrix $\mathbf{F}$ has a great degree of freedom. The above transformation matrix is chosen considering the exchange symmetry of $R(\theta_1, \theta_2)$ upon θ_1 and θ_2. The observable vector $\mathbf{S}$ then can be written as

$$\mathbf{S} = \mathbf{QF}\xi = \mathbf{D}\xi, \tag{S20}$$

where $\mathbf{D}$=$\mathbf{QF}$ is the new dictionary matrix. The coefficient vector ξ is the representation of $\mathbf{S}$ in this new dictionary, which has a good sparsity. Eq. (S20) is further solved with the LASSO (Least Absolute Shrinkage and Selection Operator) regression [18] by minimizing the l_1-norm of the coefficient vector ξ and also the difference between the reproductions and the measurements, i.e.,

$$\min_{\xi} \lambda \|\xi\|_1 + \|\mathbf{D}\xi - \mathbf{S}\|_2^2 . \tag{S21}$$

The first term in Eq. (S21) (i.e., the l_1-norm of the coefficient vector ξ) is the regular term, which represents the structure risk in the reconstruction. λ is the regularization parameter. The second term (the square error between the reproductions and the measurements) represents the empirical risk. The most commonly used linear regression algorithms are usually based on the maximum likelihood estimation, which consider only the empirical risk, and

will lead to over-fitting in the estimation when the number of parameters in the model is large (i.e., in our case). In contrast, the LASSO regression uses the maximum a posteriori probability estimation (MAP) with a Laplace priori [18] to estimate the model parameters. It takes full advantage of the sparsity of model parameters to balance regression accuracy and model complexity, and can effectively reduce the over-fitting and improve the resistance of the model to noise.

With the **R** matrix retrieved, we can then obtain the amplitude and relative phase between different θ's of the dipole moment $D(\theta)$ in terms of the derivations of Eq. (S12),

$$|D(\theta)| = \sqrt{R(\theta,\theta)}, \tag{S22}$$

$$\Phi(\theta_1) - \Phi(\theta_2) = \pm \arccos[\frac{R(\theta_1,\theta_2)}{|D(\theta_1)|\,|D(\theta_2)|}]. \tag{S23}$$

In Eq. (S23), the sign ambiguity on the right side is the due to lose of the imaginary part of $D^*(\theta_1)D(\theta_2)$ in the convolution. By assuming $\Phi(\theta)$ to be smooth, only one sign can be selected.

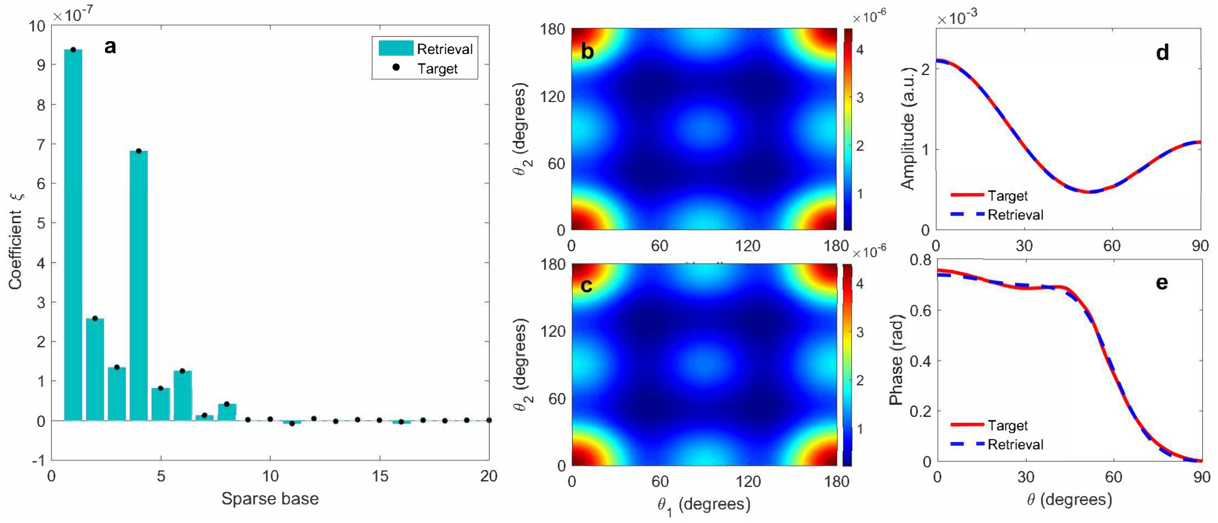

Supplementary Fig. 4: **Test of the reconstruction algorithm.** (a) Comparison between the retrieved (bars) and target (dots) coefficients ξ_i in a noise-free simulation experiment. (b) $R(\theta_1,\theta_2)$ calculated with the input dipole moment. (c) $R(\theta_1,\theta_2)$ calculated with the retrieved ξ_i in (a). (d) Comparison between the retrieved (dashed line) and input (solid line) dipole amplitude. (e) Same as (d), but for the dipole phase.

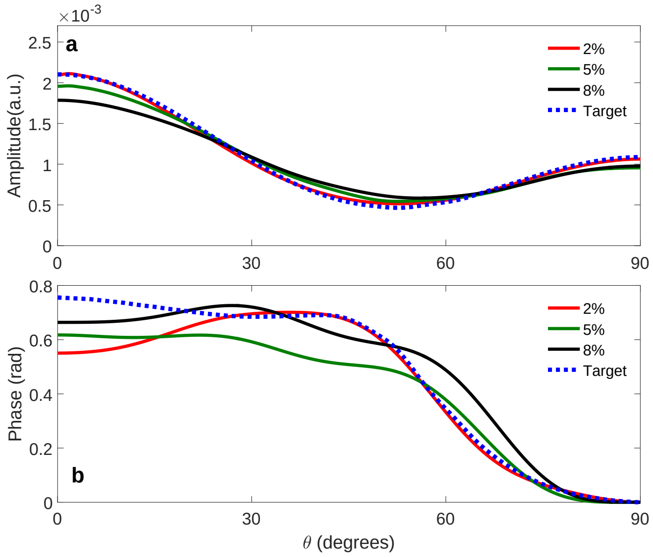

Supplementary Fig. 5: **Test of the robustness of the reconstruction algorithm against the noise.** (a) Dipole amplitude retrieved with a 2% (red solid line), 5% (green solid line), and 8% (black solid line) noise in the input signal $S(\tau)$. For comparison, the target dipole amplitude is also presented as the blue dotted line. (b) Same as (a), but for the dipole phase.

We have tested the above algorithm by using the data generated from theory. In the simulations, we first calculate the measurement $S(\tau)$ according to Eq. (S2) with a given dipole moment $D(\theta)$ and molecular axis distribution $\rho(\theta,\tau)$. Then we solve the coefficient vector ξ and retrieve the dipole moment $D(\theta)$ from $S(\tau)$ with the above algorithm. Supplementary Fig. 4 shows a comparison between the input and retrieved in an ideal (noise-free) case. One can see that the retrieved results, including the coefficients ξ_i, $R(\theta_1,\theta_2)$, and the amplitude and phase of the dipole moment $D(\theta)$, show excellent agreement with the input. Moreover, we have also checked the robustness of this algorithm by adding a gaussian noise to the input signal $S(\tau)$. Supplementary Figure 5 shows the retrieved dipole moment with the noise level of 2%, 5% and 8%, respectively. With the increase of the noise, the reconstruction accuracy is slightly reduced, but the overall agreement between the input and retrieved results still holds (at least for noise level less than 8%). In our experiment, the noise level is estimated to be less than 5%. The algorithm can be effectively applied to our experimental data.

We have also tested the above algorithm with our experimental data. Supplementary Figure 6 displays the time-dependent signals (squares) of H21 (a), H22 (b), H25 (c), and H26 (d) from N_2 molecule measured in the two-color experiment where the two-color relative

phase is zero. With the measured HHG signals, we have retrieved the single-molecule dipole moments for these harmonics with the above reconstruction algorithm. The results are shown in Supplementary Fig. 7. The retrieved angle-dependent single-molecule dipole amplitudes and phases are shown as black and red lines, respectively. With the retrieved single-molecule dipole moments, we have recalculated the time-dependent HHG signals in terms of Eq. (S2) with the alignment distribution in our experiment. As shown in Supplementary Fig. 6, our calculations (red lines) well reproduce the experimental measurements (green squares), indicating the validity of the reconstruction procedure.

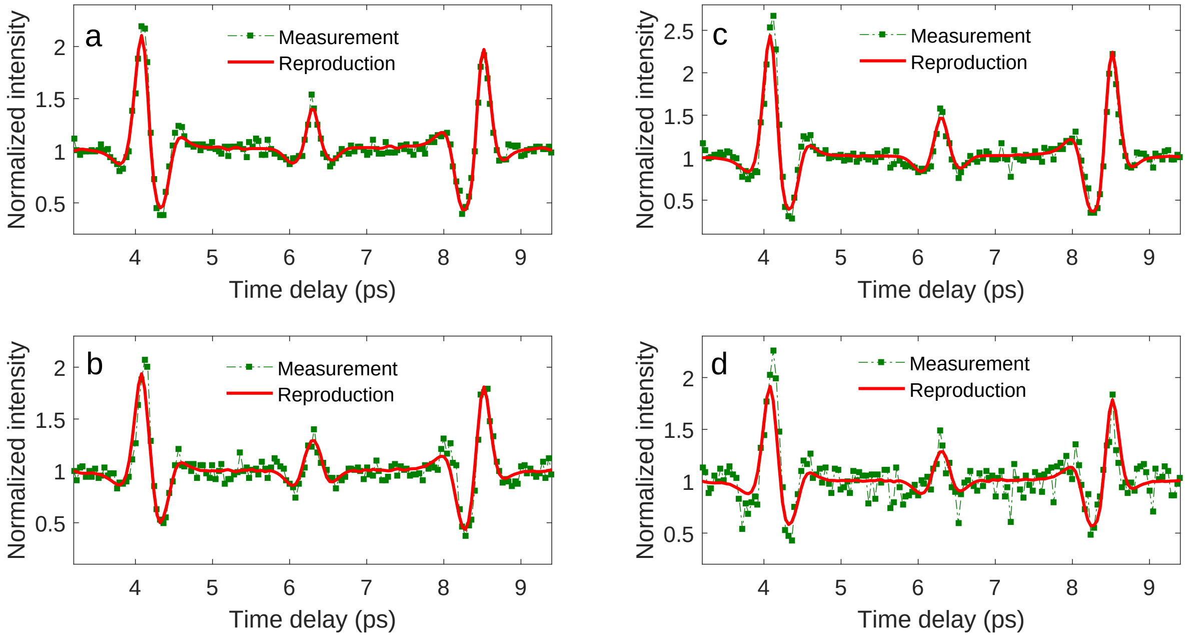

Supplementary Fig. 6: **Comparison between the reproductions and the measurements.** (a)-(d) show the reproduced (solid lines) and measured (squares) time-dependent HHG signals of H21, H22, H25, and H26 in the two-color experiment with zero relative phase, respectively.

The second step of the reconstruction is to disentangle the multichannel contributions, i.e., to retrieve the complex-valued coefficients $C_{ij}(\theta)$, from the total dipole moment $D(\theta)$ obtained in Step 1. To this end, we have performed experiment with a two-color laser field. The retrieved $D(\theta)$ at different relative phases α provide a set of equations for the reconstruction, i.e.,

$$D(\theta;\alpha) = \sum_{ij} C_{ij}(\theta)\bar{D}_{ij}(\theta;\alpha). \tag{S24}$$

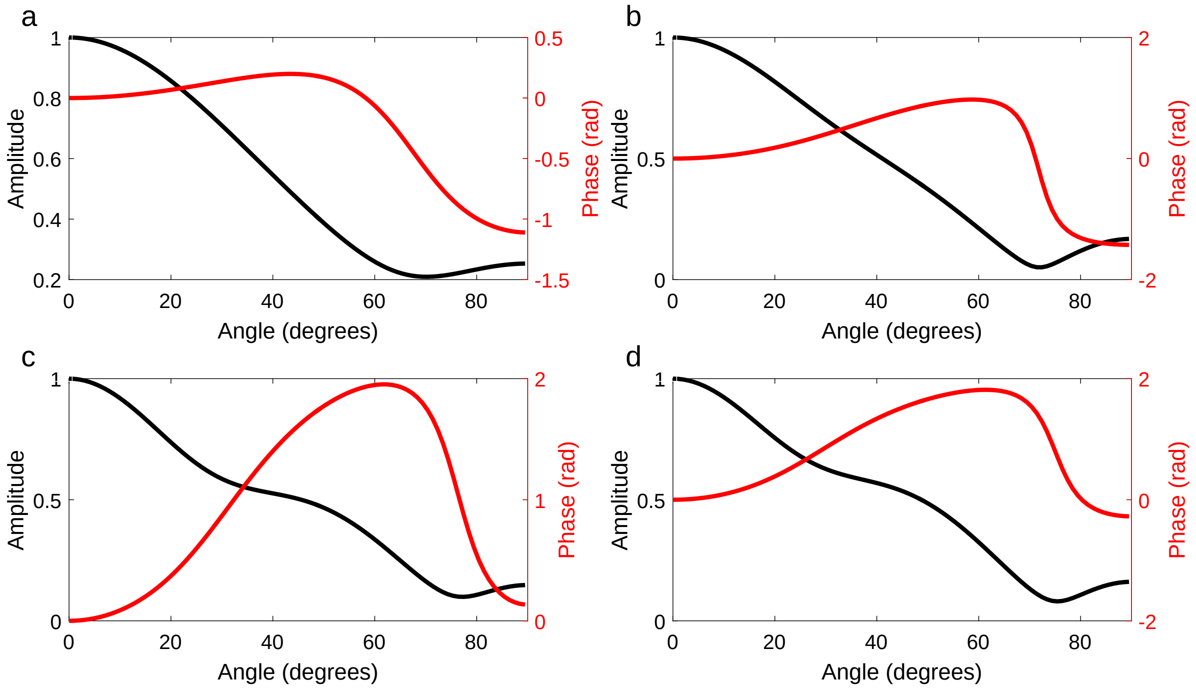

Supplementary Fig. 7: **Retrieved single-molecule dipole moments.** (a) Angle-dependent single-molecule dipole amplitudes (black line) and phases (red line) of H21 of N_2 retrieved from the time-dependent HHG signals measured in the two-color experiment with zero relative phase. (b)-(d) Same as (a), but for H22, H25, and H26, respectively.

Since the SH field in our two-color experiment is weak enough and hardly alters the laser-induced electron dynamics, the coefficients $C_{ij}(\theta)$ can be assumed to be independent of the relative phase α (see Supplementary Note 5 below). In our reconstruction, $\bar{D}_{ij}(\theta;\alpha)$ is calculated under the experimental laser conditions in terms of Eq. (S10). In the calculations, the exact scattering wave is employed to calculate the transition dipoles. Given that the phase retrieved in Step 1 is the relative phase between different alignment angles, which may have a global phase difference from the actual dipole phase. Here we divide Eq. (S24) by considering the dipole amplitude and relative phase separately, i.e.,

$$|D(\theta;\alpha)| = \left|D^{\otimes}(\theta;\alpha)\right|, \tag{S25}$$

$$\Phi(\theta;\alpha) - \Phi(0;\alpha) = \Phi^{\otimes}(\theta;\alpha) - \Phi^{\otimes}(0;\alpha), \tag{S26}$$

where

$$D^{\otimes}(\theta;\alpha) = \sum_{ij} C_{ij}(\theta)\bar{D}_{ij}(\theta;\alpha) = \left|D^{\otimes}(\theta;\alpha)\right| \exp[i\Phi^{\otimes}(\theta;\alpha)]. \tag{S27}$$

Note that, in our reconstruction, only the most relevant ionic states ψ_j (that is, $\tilde{X}$ and $\tilde{A}$ states for N_2, and $\tilde{X}$, $\tilde{A}$, and $\tilde{B}$ states for CO_2) are included. The number of relative phases α (14 sets) used in our experiment is greater than that of the coefficients $C_{ij}(\theta)$. Thus, Eqs. (S25)-(S26) are overdetermined equations. The coefficients $C_{ij}(\theta)$ can be obtained by solving Eqs. (S25)-(S26) with the genetic algorithm.

With the coefficients $C_{ij}(\theta)$ retrieved, the wave function of the molecular ion at the recombination instant can be written as

$$\psi_{\mathrm{sum}} = \sum_{ij} \gamma_i(\theta) C_{ij}(\theta) \psi_j, \tag{S28}$$

where $\gamma_i(\theta)$ is the initial population of the state i, which is related to the alignment-angle-dependent ionization rate $\eta_i(\theta)$ by $|\gamma_i(\theta)|^2 = \eta_i(\theta)/\Sigma_i \eta_i(\theta)$. In our reconstruction, the alignment-angle-dependent ionization rates $\eta_i(\theta)$ of different molecular orbitals are calculated with the MO-ADK theory [19]. In our reconstruction, the ionization-induced initial phases of the ionic states are contained in the retrieved coefficients $C_{ij}(\theta)$. The population coefficient of the molecular orbital ψ_j of the molecular ion at the recombination instant is then given by

$$p_j = \sum_{i} C_{ij}(\theta) \gamma_i(\theta). \tag{S29}$$

Repeating the above procedure for different harmonic orders, we can then achieve a temporal measurement of ultrafast charge migration in the molecules.

Supplementary Note 5: Time-dependent density functional theory calculations

To evaluate the evolution of a molecular system in the external laser field, we have performed three-dimensional simulations based on the time-dependent density functional theory (TDDFT). In the TDDFT framework, the molecular system is described by a series of one-particle Kohn-Sham (KS) orbitals, of which the evolution can be obtained by solving the time-dependent Kohn-Sham (TDKS) equations [20]

$$i\frac{\partial \psi_i(\mathbf{r},t)}{\partial t} = \left[-\frac{\nabla^2}{2} + V_{\mathrm{KS}}(\mathbf{r},t)\right] \psi_i(\mathbf{r},t), \tag{S30}$$

where

$$V_{\mathrm{KS}}(\mathbf{r},t) = V_{\mathrm{L}}(\mathbf{r},t) + V_{\mathrm{H}}(\mathbf{r},t) + V_{\mathrm{xc}}(\mathbf{r},t) + V_{\mathrm{ion}}(\mathbf{r},t). \tag{S31}$$

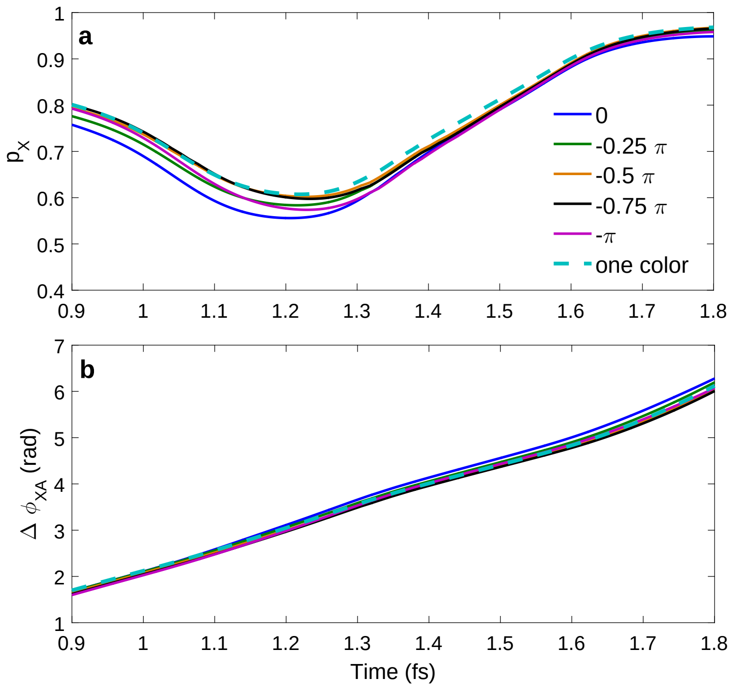

Supplementary Fig. 8: **Electron dynamics in the two-color field.** (a) TDDFT calculations of the time-dependent population amplitudes of the $\tilde{X}$ ($|p_{\mathrm{X}}|$) state of N_2^+ ion for different relative phases of the two-color laser fields (solid lines). For comparison, the result calculated with the fundamental pulse alone is also presented (dashed line). (b) Same as (a), but for the relative phase between the wave functions of $\tilde{X}$ and $\tilde{A}$ states ($\Delta\phi_{\mathrm{XA}}$) of the N_2^+ ion.

Here, the first term $V_{\mathrm{L}}(\mathbf{r},\ t)$ represents the interaction of the electrons with the laser field. The second term V_{H} $(\mathbf{r},\ t)$ is the time-dependent Hartree potential describing the interaction between electrons. The third term V_{xc} $(\mathbf{r},\ t)$ is the exchange correlation potential, for which we chose the generalized gradient approximation (GGA) proposed in [21]. The last term $V_{\mathrm{ion}}(\mathbf{r},\ t)$ denotes the electron-ion interactions, which is described by norm-conserving Troullier-Martins pseudopotentials [22] in the Kleinman-Bylander form [23]. The TDKS equations are discretized and solved with the OCTOPUS package [24]. The inclusion of laser-induced coupling between different orbitals in the TDKS equations allows us to simulate the evolution of different orbitals during the driving laser pulse. In our calculations, the transition amplitude C_{ij} is given by the projection of the TDKS orbital $\psi_i(\mathbf{r}, t)$ onto the initial KS oribtal $\psi_j(\mathbf{r}, 0)$, i.e., $C_{ij} = \langle\psi_j(\mathbf{r}, 0)|\psi_i(\mathbf{r}, t)\rangle$. The amplitude of each ion state is then calculated according to Eq. S29.

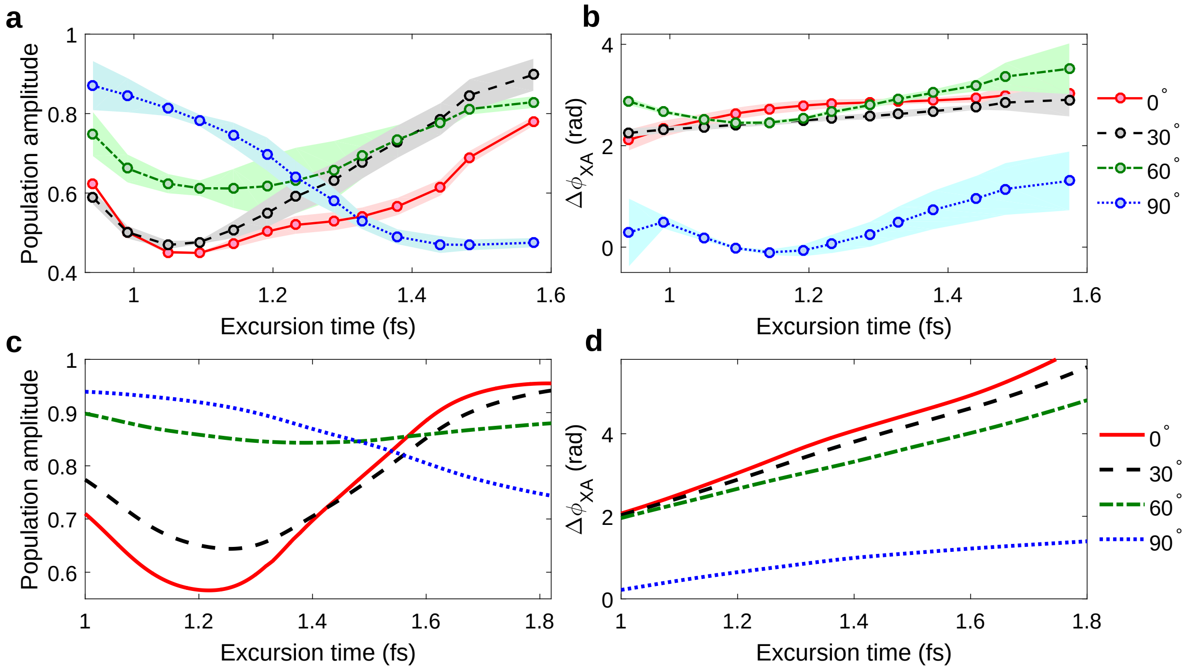

Supplementary Fig. 9: **Comparison between the reconstructions and TDDFT calculations of N_2.** (a)-(b) Reconstructed population amplitude of the $\tilde{X}$ state (a) and the relative phase between the wave functions of $\tilde{X}$ and $\tilde{A}$ state (b) for the alignment angles of 0° (red circles), 30° (black circles), 60° (green circles), and 90° (blue circles), respectively. (c)-(d) Same as (a)-(b), but for the TDDFT calculations.

Here, we first select N_2 as an example to demonstrate the influence of the two-color laser field on the electron dynamics in the ionized molecules. To calculate the sub-cycle electron dynamics, the two-color fields used in our simulations are expressed as $E(t) = E_1 \cos(\omega t) + E_2 \cos(2\omega t + \alpha)$. Here E_1 and E_2 are the laser intensities of the two-color fields, which are taken from the experiment. α is the relative phase between the two-color fields. Supplementary Figure 8(a) shows the time-dependent population amplitudes of the $\tilde{X}$ state of N_2^+ ion calculated with different relative phases of the two-color laser field. Supplementary Figure 8(b) shows the corresponding relative phases between the wave functions of $\tilde{X}$ and $\tilde{A}$ states. For comparison, the results calculated with the fundamental pulse alone are also presented by the dashed lines. One can see that, the calculations (both the population amplitudes of the $\tilde{X}$ state and the relative phases between the wave functions of $\tilde{X}$ and $\tilde{A}$ states) at different relative phases of the two-color field are very close to that with the fundamental pulse alone, indicating negligible influence of the weak SH field on the electron

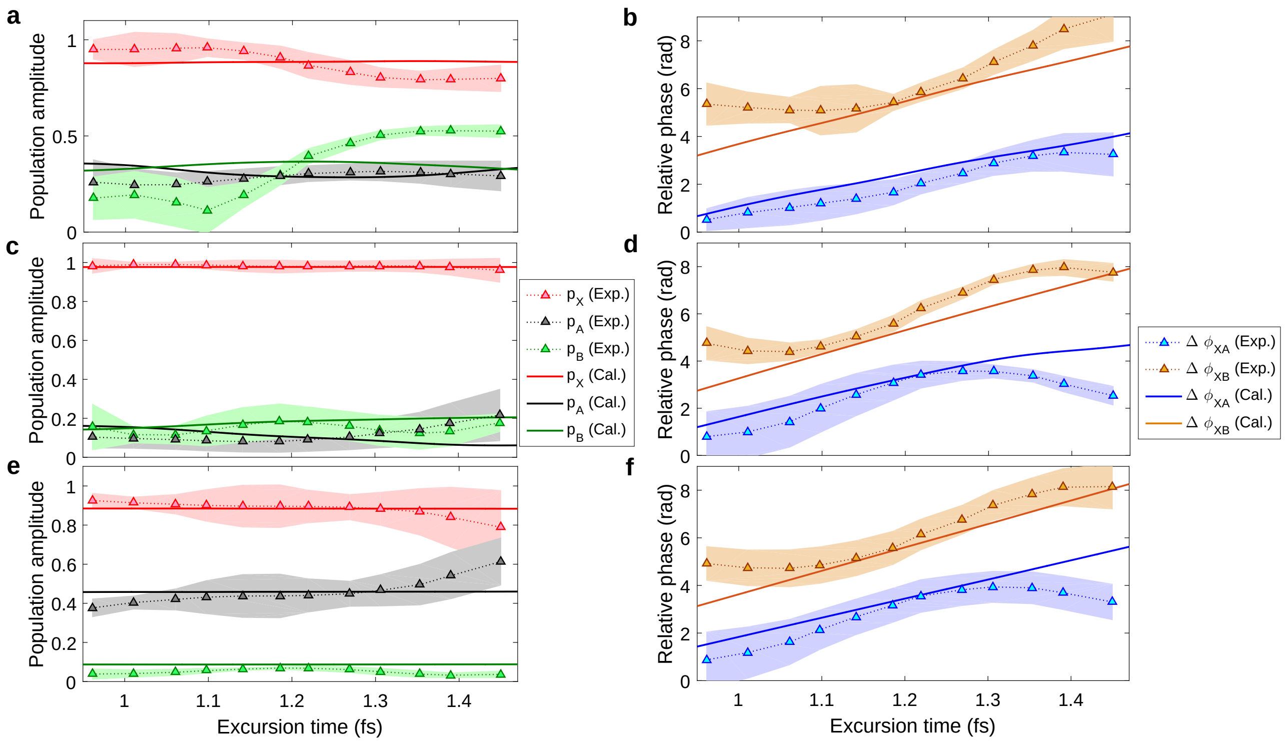

Supplementary Fig. 10: **Comparison between the reconstructions and TDDFT calculations of CO_2.** (a) Reconstructed population amplitudes of the $\tilde{X}$ (red triangles), $\tilde{A}$ (black triangles), and $\tilde{B}$ (green triangles) states for the alignment angles of 0°. Red, black, and green lines are the corresponding calculation results. (b) Reconstructed relative phases between the wave functions of $\tilde{X}$ and $\tilde{A}$ states (blue triangles) and $\tilde{X}$ and $\tilde{B}$ states (yellow triangles). Blue and yellow lines are the corresponding calculation results. (c)-(d), (e)-(f) Same as (a)-(b), but for the alignment angles of 45° and 90°, respectively.

dynamics underlying the HHG process. In the following, we use the calculations with the fundamental pulse alone to evaluate our experimental reconstructions.

In Supplementary Fig. 9, we compare the population amplitude and relative phase from the reconstructions [(a)-(b)] and from TDDFT calculations [(c)-(d)] of N_2 for different alignment angles. As shown in Supplementary Fig. 9(a), for small alignment angles, e.g., $\theta = 0°$ (red circles) and 30° (black circles), the population of the $\tilde{X}$ state decreases for the early excursion times after ionization and reaches a minimum at t=1.05 fs before increasing again. Such time dependence of the population of the $\tilde{X}$ state agrees with the TDDFT calculations (red solid and black dashed lines) presented in Supplementary Fig. 9(c), but by a $\sim$150 as

difference in the location of the minimum. This deviation may be attributed to the approximation of electron correlation in TDDFT calculations. For the larger alignment angles, e.g., $\theta = 60°$ [green circles in Supplementary Fig. 9(a)], the time dependence of the population becomes weaker. For $\theta = 90°$ [blue circles in Supplementary Fig. 9(a)], the retrieved population of $\tilde{X}$ state decreases monotonically with the excursion time. For the relative phase between the wave functions of $\tilde{X}$ and $\tilde{A}$ states, the retrieved results for $\theta = 0°$, 30° and 60° are all close to each other, while for $\theta = 90°$ [see Fig. 4(b)], the phase is much smaller. All these reconstructions are consistent with those obtained from TDDFT calculations.

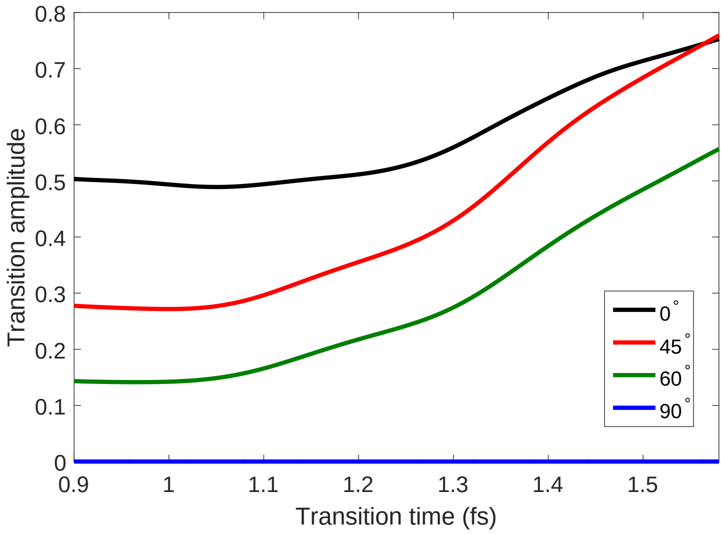

Supplementary Fig. 11: **TDDFT calculations of the time-dependent transition amplitudes from the $\tilde{X}$ state to $\tilde{A}$ state in CO_2^+ ion for different molecular alignment angles.** Here, the calculations are performed with the fundamental pulse alone. The transition probability is shown to decrease gradually with the increase of molecular alignment angle.

We have also performed TDDFT calculations for CO_2 molecule. The results are shown in Supplementary Fig. 10 (solid lines). For comparison, the experimental reconstructions are also presented as the triangles. As shown in the left column of Supplementary Fig. 10, the population of the $\tilde{B}$ state (green triangles) decreases gradually as molecular alignment angle increases, because of the preferential ionization of HOMO-2 orbital of CO_2 (therefore the large population of $\tilde{B}$ state) at small molecular alignment angles. For the $\tilde{A}$ state, it shows a considerable population at the alignment angle of $\theta = 0°$ [black triangles in Supplementary

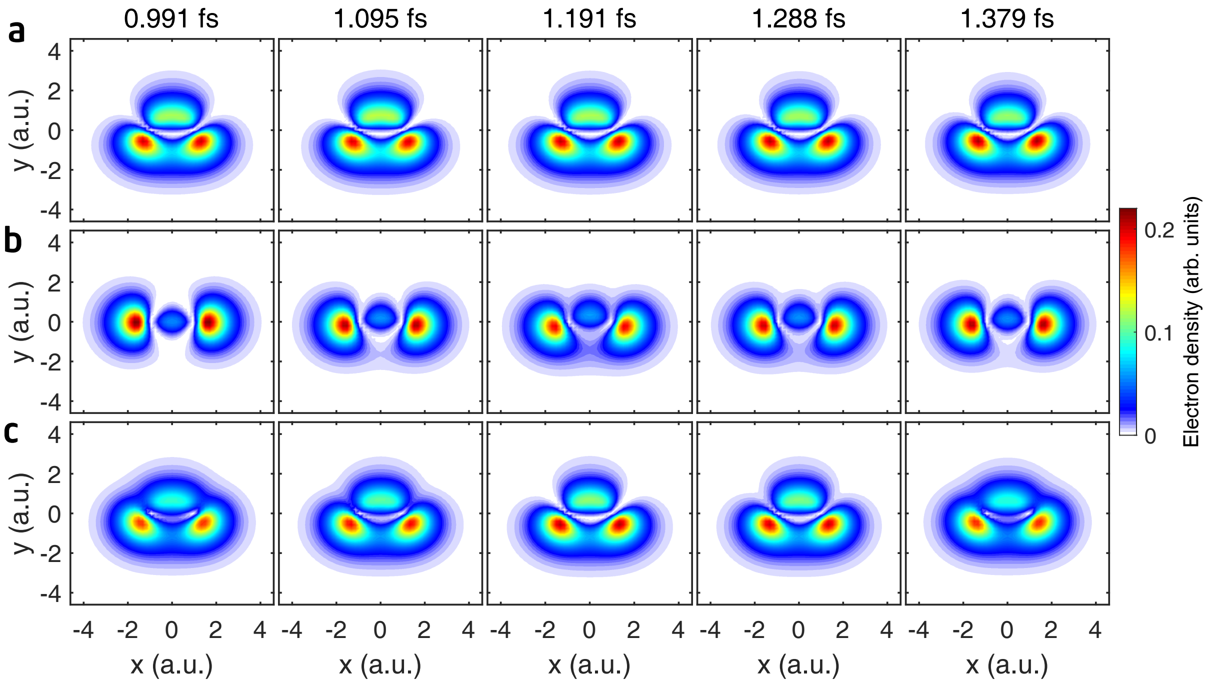

Supplementary Fig. 12: **Snapshots of the reconstructed hole densities in N_2^+ for the alignment angle of 0 degree.** (a) is the results reconstructed with the retrieved single-molecule dipole moments. (b) same as (a), but for the reconstruction with the angle-averaged harmonic dipole moments in our experiment. (c) plots the TDDFT results for comparison.

Fig. 10(a)]. Generally, the population of the $\tilde{A}$ state of CO_2 is not appreciable at $\theta = 0°$ due to the weak ionization rate of the HOMO-1 at this angle. Our TDDFT calculations suggest that the population of $\tilde{A}$ state at $\theta = 0°$ mainly arises from the nonadiabatic transition from the $\tilde{X}$ state (see Supplementary Fig. 11). The coupling between $\tilde{A}$ and $\tilde{X}$ states modifies their populations as the excursion time changes. Moreover, this transition amplitude is shown to decrease with the increase of molecular alignment angles (see Supplementary Fig. 11). From Supplementary Figs. 9 and 10, we can see reasonable agreement between the reconstructed data and the ones from the TDDFT calculations.

Supplementary Note 6: Influence of alignment average on the reconstruction

In this section, we demonstrate the influence of alignment average on the reconstruction. We first calculate the angle-averaged harmonic dipoles in terms of Eq.(S1) by using the single-molecule dipole moments retrieved from our two-color experiment. The molec-

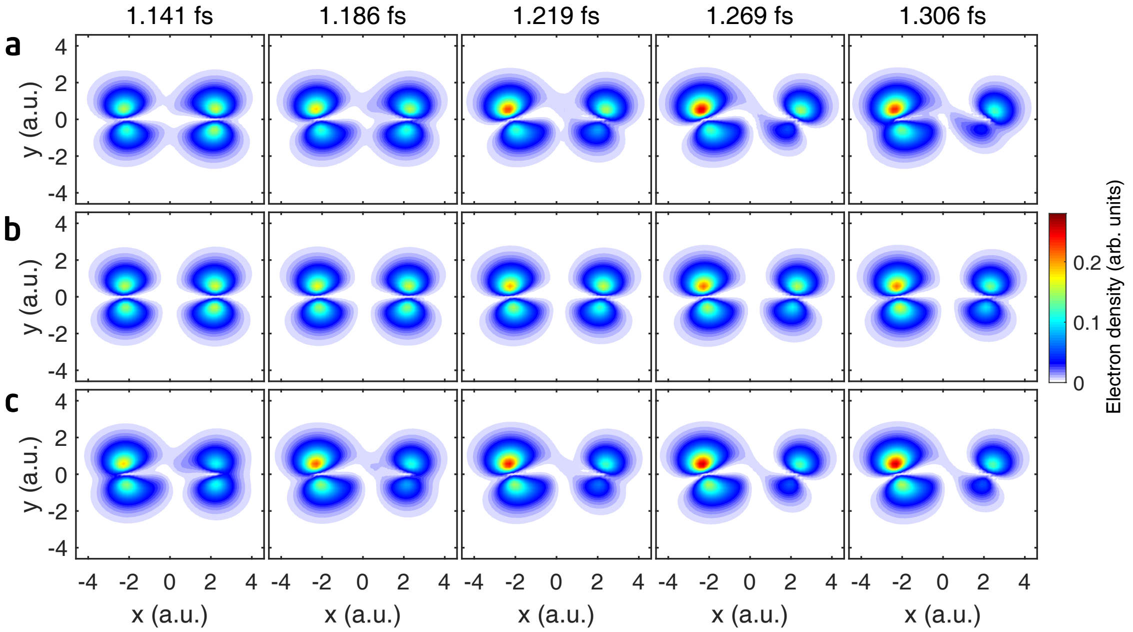

Supplementary Fig. 13: **Snapshots of the reconstructed hole densities in CO_2^+ for the alignment angle of 0 degree.** (a) is the results reconstructed with the retrieved single-molecule dipole moments. (b) same as (a), but for the reconstruction with the angle-averaged harmonic dipole moments in our experiment. (c) plots the TDDFT results for comparison.

ular axis distribution $\rho(\theta)$ at the maximum alignment in our experiment is used in the calculation. With the angle-averaged harmonic dipoles, we have reconstructed the electron dynamics with the procedure introduced in Supplementary Note 4. As an example, we have plotted the results of N_2 at the alignment angle of 0 degree in Supplementary Fig. 12. Supplementary Fig. 12(a) shows some selected snapshots of the hole densities reconstructed with the retrieved single-molecule dipoles. Supplementary Fig. 12(b) is the corresponding results reconstructed with the angle-averaged dipoles. For comparison, we have also plotted the TDDFT results in Supplementary Fig. 12(c). It's evident that the hole dynamics reconstructed with the angle-averaged dipoles are very different from that with the single-molecule dipoles and show worse agreement with the TDDFT results. This difference is mainly due to the underestimated contribution of the HOMO-1 orbital of N_2 molecule in the angle-averaged dipoles. Such a result indicates a severe influence of the angular averaging on the accuracy of the reconstruction. Note that, we have also checked the influence of the alignment degree on the reconstruction. We find that slight increase in the alignment

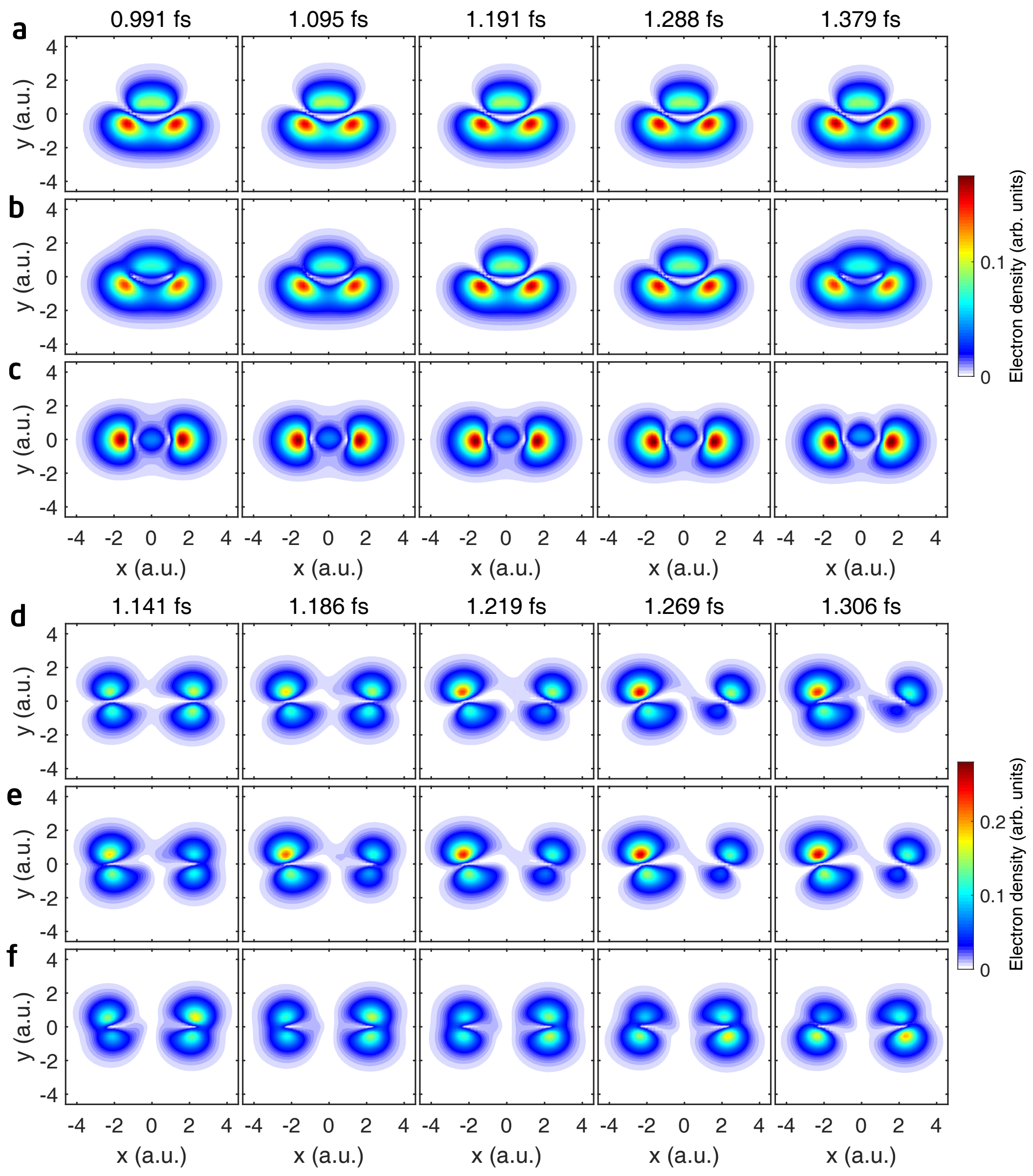

Supplementary Fig. 14: **Comparison between the laser-driven and field-free charge migrations.** (a) Experimentally reconstructed and (b) TDDFT simulated charge migration in N_2^+ for the alignment angle of 0 degree. (c) Same as (b), but for the simulated field-free results. (d)-(f) Same as (a)-(c), but for the results of CO_2^+ at the alignment angle of 0 degree.

degree, e.g., $\langle \cos^2 \theta \rangle = 0.65$, does not improve the accuracy of the reconstruction much. We have also performed similar reconstructions for CO_2^+. The results are shown in Supplementary Fig. 13. One can also see significant influence of the alignment average on the reconstruction.

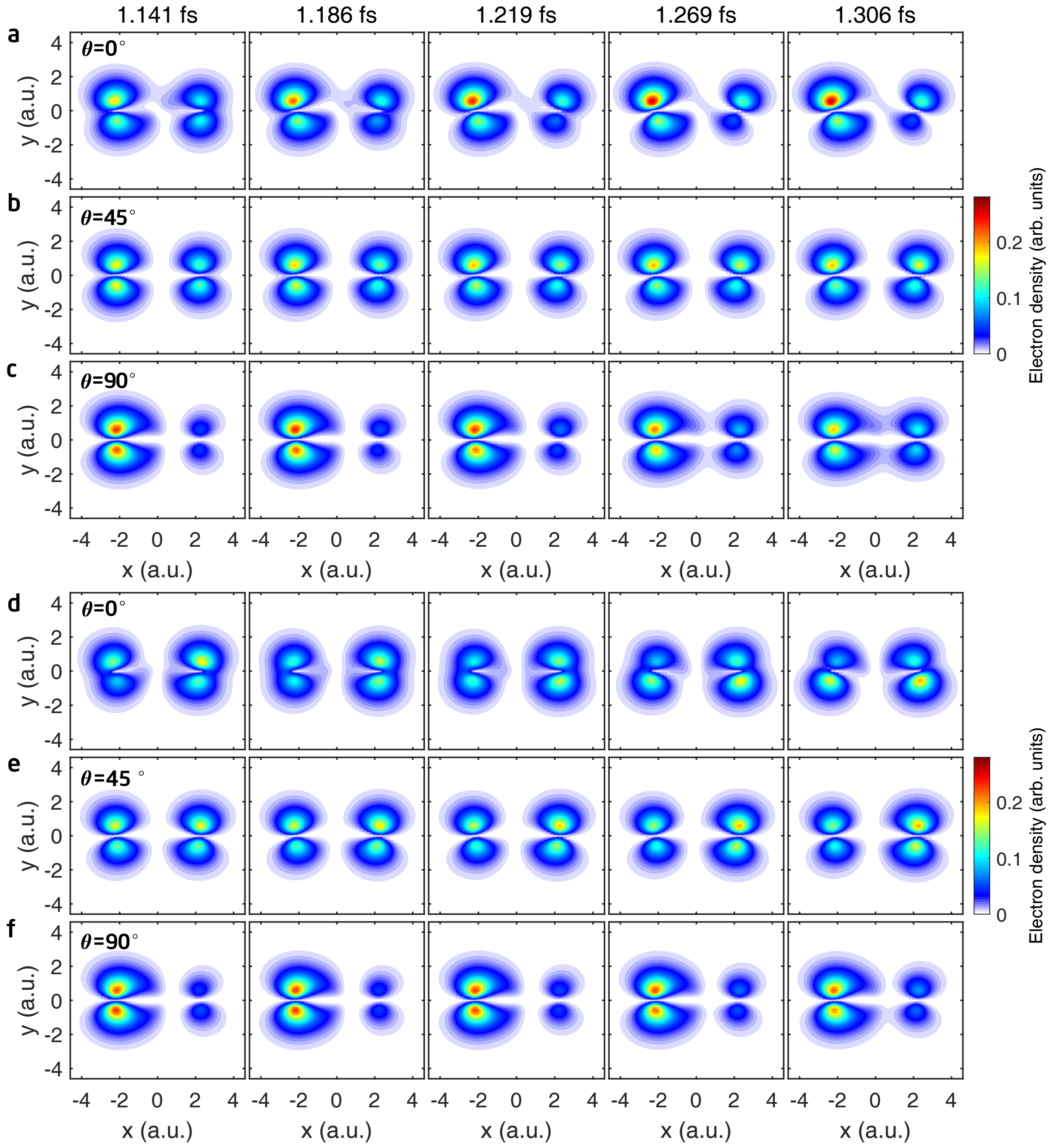

Supplementary Fig. 15: **Alignment-dependence of the field-free charge migration.** (a)-(c) TDDFT simulations of the laser-driven charge migration in CO_2^+ for the alignment angle of 0, 45, and 90 degree, respectively. (d)-(f) Same as (a)-(c), but for the simulations of the field-free results.

Supplementary Note 7: Field-free charge migration in N_2^+ and CO_2^+

In this section, we discuss the field-free charge migrations in N_2^+ and CO_2^+. As mentioned in the main text, in the HHG process, the electron dynamics will be influenced by the intense laser field. Thus charge migration derived from HHS is generally different from the

original field-free charge migration defined by Cederbaum *et al.* [25]. From Supplementary Fig. 14, one can see that the reconstructed hole densities for both N_2^+ and CO_2^+ at the alignment angle of 0° [(a),(d)] agree well with the TDDFT simulations [(b),(e)]. Thus, it's reasonable to evaluate the field-free charge migration in the two molecules with the corresponding TDDFT results. In Supplementary Figs. 14 (c) and (f), we have simulated the field-free hole dynamics for these two molecules. Compared to the laser-driven results (both experiment and theory), one can see considerable influence of the laser field on the electron dynamics. We have also studied the alignment-dependence of the field-free charge migration. The field-free TDDFT simulation results of CO_2 for the alignment angle of 0°, 45°, 90° are shown in Supplementary Figs. 15(d)-(f) as the example. For comparison, the simulated laser-driven results are also presented in Supplementary Figs. 15(a)-(c). One can see that laser control over molecular charge migration depends sensitively on the molecular alignment.

Supplementary references

[1] L. He *et al.*, Spectrally resolved spatiotemporal features of quantum paths in high-order-harmonic generation, *Phys. Rev. A* **92**, 043403 (2015).

[2] A. T. Le, R. R. Lucchese, S. Tonzani, T. Morishita, and C. D. Lin, Quantitative rescattering theory for high-order harmonic generation from molecules, *Phys. Rev. A* **80**, 013401 (2009).

[3] C. D. Lin, A. T. Le, Z. Chen, T. Morishita, and R. Lucchese, Strong-field rescattering physics—self-imaging of a molecule by its own electrons, *J. Phys. B: At. Mol. Opt. Phys.* **43**, 122001 (2010).

[4] O. Smirnova, Y. Mairesse, S. Patchkovskii, N. Dudovich, D. Villeneuve, P. Corkum, and M. Y. Ivanov, High harmonic interferometry of multi-electron dynamics in molecules, *Nature* **460**, 972 (2009).

[5] O. Smirnova, S. Patchkovskii, Y. Mairesse, N. Dudovich, and M. Y. Ivanov, Strong-field control and spectroscopy of attosecond electron-hole dynamics in molecules, *Proc. Natl. Acad. Sci. U.S.A.* **106**, 16556 (2009).

[6] L. He *et al.*, Real-time observation of molecular spinning with angular high-harmonic spectroscopy, *Phys. Rev. Lett.* **121**, 163201 (2018).

[7] Y. He *et al.*, Direct imaging of molecular rotation with high-order-harmonic generation, *Phys. Rev. A* **99**, 053419 (2019).

[8] H. Stapelfeldt and T. Seideman, Colloquium: Aligning molecules with strong laser pulses, *Rev. Mod. Phys.* **75**, 543557 (2003).

[9] C. P. Koch, M. Lemeshko, and D. Sugny, Quantum control of molecular rotation, *Rev. Mod. Phys.* **91**, 035005 (2019).

[10] X. Wang, A. Le, Z. Zhou, H. Wei, and C. D. Lin, Theory of retrieving orientation-resolved molecular information using time-domain rotational coherence spectroscopy, *Phys. Rev. A* **96**, 023424 (2017).

[11] B. Wang *et al.*, Retrieval of full angular-and energy-dependent complex transition dipoles in the molecular frame from laser-induced high-order harmonic signals with aligned molecules, *Phys. Rev. A* **101**, 063417 (2020).

[12] R. Rubinstein, A. M. Bruckstein, and M. Elad, Dictionaries for sparse representation

modeling, *Proceedings of the IEEE* **98**, 1045-1057 (2010).

[13] A. Beck and M. Teboulle, A fast iterative shrinkage-thresholding algorithm for linear inverse problems, *SIAM J. Imaging Sci.* **2**, 183-202 (2009).

[14] J. Yang, Z. Wang, Z. Lin, S. Cohen, and T. Huang, Coupled dictionary training for image super-resolution, *IEEE transactions on image processing.* **21**, 3467-3478 (2012).

[15] V. M. Patel and R. Chellappa, Sparse representations and compressive sensing for imaging and vision, Springer Science & Business Media (2013).

[16] S. Rudy, S. Brunton, J. L. Proctor, and J. N. Kutz, Data-driven discovery of partial differential equations, *Sci. Adv.* **3**, e1602614 (2017).

[17] J. Wright *et al.*, Sparse representation for computer vision and pattern recognition, *Proceedings of the IEEE* **98**, 1031-1044 (2010).

[18] L. Meier, D. Van, and P. Buhlmann, The group lasso for logistic regression, *Journal of the Royal Statistical Society B.* **70**, 53-71 (2008).

[19] X. M. Tong, Z. X. Zhao, and C. D. Lin, Theory of molecular tunneling ionization, *Phys. Rev. A* **66**, 033402 (2002).

[20] E. Runge, and E. Gross, Density-functional theory for time-dependent systems, *Phys. Rev. Lett.* **52**, 997 (1984).

[21] J. P. Perdew, K. Burke, and M. Ernzerhof, Generalized gradient approximation made simple, *Phys. Rev. Lett.* **77**, 3865 (1996).

[22] N. Troullier and J. L. Martins, Efficient pseudopotentials for plane-wave calculations, *Phys. Rev. B* **43**, 1993 (1991).

[23] L. Kleinman and D. M. Bylander, Efficacious form for model pseudopotentials, *Phys. Rev. Lett.* **48**, 1425 (1982).

[24] M. Marques, A. Castro, G. F. Bertsch, and A. Rubio, octopus: a first-principles tool for excited electronion dynamics, *Comput. Phys. Commun.* **151**, 60-78 (2003).

[25] L. S. Cederbaum and J. Zobeley, Ultrafast charge migration by electron correlation, *Chem. Phys. Lett.* **307**, 205-210 (1999).
