# Supplementary material for: Filming movies of attosecond charge migration in single molecules with high harmonic spectroscopy
Source: Nat Commun. 2022 Aug 6;13:4595. doi: 10.1038/s41467-022-32313-0 (PMC9357086; doi:10.1038/s41467-022-32313-0)
Supplement: Supplementary file 3 — Description of Additional Supplementary Files [file 41467_2022_32313_MOESM3_ESM.pdf]

Description of Additional Supplementary Files

File name: Supplementary Movie 1

Description: Reconstructed charge migration in CO2 molecule for the alignment angle of 0 degree.

File name: Supplementary Movie 2

Description: Reconstructed charge migration in CO2 molecule for the alignment angle of 15 degrees.

File name: Supplementary Movie 3

Description: Reconstructed charge migration in CO2 molecule for the alignment angle of 45 degrees.

File name: Supplementary Movie 4

Description: Reconstructed charge migration in CO2 molecule for the alignment angle of 90 degrees.

File name: Supplementary Movie 5

Description: Reconstructed charge migration in N2 molecule for the alignment angle of 0 degree.

File name: Supplementary Movie 6

Description: Reconstructed charge migration in N2 molecule for the alignment angle of 30 degrees.

File name: Supplementary Movie 7

Description: Reconstructed charge migration in N2 molecule for the alignment angle of 60 degrees.

File name: Supplementary Movie 8

Description: Reconstructed charge migration in N2 molecule for the alignment angle of 90 degrees.
